# Elevated Free Thyroxine Levels Are Associated with Poorer Overall Survival in Patients with Gastroesophageal Cancer: A Retrospective Single Center Analysis

**DOI:** 10.1007/s12672-019-00374-1

**Published:** 2019-12-28

**Authors:** H. C. Puhr, P. Wolf, A. S. Berghoff, S. F. Schoppmann, M. Preusser, Aysegul Ilhan-Mutlu

**Affiliations:** 1 Department of Medicine I, Division of Oncology, Medical University of Vienna, Waehringer Guertel 18-20, A1090 Wien, Austria; 2 Comprehensive Cancer Center Vienna, Upper-GI Tumors Unit, Medical University of Vienna, Waehringer Guertel 18-20, A1090 Wien, Austria; 3 Department of Internal Medicine III, Division of Endocrinology and Metabolism, Medical University of Vienna, Waehringer Guertel 18-20, A1090 Wien, Austria; 4 Department of Surgery, Medical University of Vienna, Waehringer Guertel 18-20, A1090 Wien, Austria

**Keywords:** Gastric, Esophagus, Gastroesophageal, Hyperthyroidism, Thyroxine

## Abstract

As endocrinological parameters such as thyroid hormones modulate proliferative, metabolic, and angiogenic pathways, it is surmised that their levels can be associated with cancer development and progression. Most patients with gastroesophageal cancer are diagnosed very late and have a poor prognosis, yet the association with endocrinological parameters has not been addressed so far. The aim of this study was to correlate hormones with the outcome, so new prognostic and potentially therapeutic markers can be defined. We analyzed clinical and endocrinological parameters including history of thyroid disorders and laboratory analyses of thyroid hormones and correlated these with the overall survival in a large European cohort of patients with inoperable locally advanced or metastatic gastroesophageal cancer treated between 2002 and 2018 at the Vienna General Hospital, Austria. In total, the survival outcome of 258 patients was evaluated. Higher levels of fT4 (*p* = 0.041, HR = 2.202) and lower levels of T3 (*p* = 0,003, HR = 0,141) were associated with significantly shorter survival. However, the overall survival of patients with known thyroid disorders did not differ significantly from euthyroid patients (euthyroid, 283 days; hyperthyroid, 354 days; hypothyroid, 284 days; *p* = 0.472). Elevated fT4 levels are associated with poorer overall survival of patients with gastroesophageal cancer in advanced stages. Since data on the correlation of endocrinological parameters and gastroesophageal cancer are scarce, this analysis is an important impulse for further studies concerning the impact of thyroxine on patients with cancer of the upper GI tract.

## Introduction

Cancer of the upper gastrointestinal (GI) tract which encompasses esophageal cancer, gastric cancer, as well as cancer of the gastroesophageal junction is a frequent disease and major contributor to global disease burden. Worldwide, cancer of the upper GI tract is the second leading cause of cancer-related death, with about 1.4 million newly diagnosed cases (951,000 cases of gastric cancer and 456,000 cases of esophageal cancer) in 2012 [1].

Gastroesophageal cancer is usually asymptomatic in early stages, and symptoms such as weight loss, dysphagia, dyspepsia, vomiting, early satiety, and/or iron deficiency anemia develop mostly in advanced tumor stages [2, 3]. In consequence, most patients in the western world are diagnosed very late during the course of the disease, at a locally advanced (stage III) or metastatic stage (stage IV). Even though the survival showed a steady increase during the past decades independent of the tumor stage, the prognosis remains poor especially in more advanced stages. For esophageal and gastric cancer, 5-year relative survival is 23.6 and 30.6% at locally advanced stages and 4.8 and 5.2% at metastatic stages, respectively [4, 5]. Knowledge on factors that support this unfavorable outcome is scarce, yet desperately needed to improve it. Thus, biological factors, which drive tumor progression and metastasis, are of high clinical interest for the development of new targeted therapeutic approaches in order to potentially prevent or slow down the tumor growth.

Thyroid hormones modulate proliferative, metabolic, and angiogenic pathways, and previous studies suggest that their levels can be associated with cancer development and progression [6–9]. Also, loss of normal functions of thyroid receptors might also contribute to cancer development, progression, and metastasis [10, 11]. However, data on endocrinological parameters and their correlation with cancer incidence as well as progression, especially in the field of gastrointestinal oncology, are scarce.

Thus, the aim of this study was to investigate the prognostic impact of thyroid hormones in a large, well-defined cohort of patients suffering from metastatic gastroesophageal cancer.

## Patients and Methods

### Patient Collection

Patients with histologically proven advanced or metastatic (stage IV) gastroesophageal cancer treated between March 2002 and June 2018 were identified from the patient database of the Vienna General Hospital, Austria. Clinical information including patient demographics, therapy regimens, thyroid hormones, and survival outcome was obtained.

All patients underwent tumor staging prior to therapy according to the local hospital standard, including history taking, physical examination, routine hematologic tests, upper gastrointestinal endoscopy with histological biopsy, and computed tomography of the chest and abdomen.

Patients were treated according to the individual decision of an interdisciplinary tumor board, which ensured the best possible treatment according to the respective standard of knowledge at the time of diagnosis. As all treatments were in a palliative setting due to the advanced tumor stage, the prolongation of OS and the reduction of symptoms were the main goals.

The treatment included systemic (immuno)chemotherapy and/or palliative gastrectomy and/or radiation therapy of the primary tumor, lymph nodes, or metastatic sites. Patients with neoadjuvant treatment of the same tumor in an initially curative setting were excluded from the study.

Routine re-evaluation of the tumor status was performed at least every 3 months with computed tomography or magnetic resonance imaging. Evaluation of the response was done according to the current RECIST criteria by experienced radiologists [12].

Patients were followed up until death according to the hospital or public records or loss to follow-up.

All procedures followed were in accordance with the ethical standards of the responsible committee on human experimentation and with the Helsinki Declaration of 1964 and later versions. Due to the retrospective design, no separate informed consent was necessary in the scope of this study. The study was approved by the ethics committee of the Medical University of Vienna.

### Recruitment of Endocrinological Parameters and Disorders

We evaluated endocrinological parameters including thyroid hormones including thyroid-stimulating hormone (TSH), triiodothyronine (T3), thyroxine (T4), free triiodothyronine (fT3), and free thyroxine (fT4). All parameters were determined using standard assays and procedures according to hospital routine. The normal ranges of parameters are TSH, 0.44–3.77 μU/ml; T3, 0.8–1.8 ng/ml; T4, 58–124 ng/ml; fT4, 0.76–1.66 ng/dl; and fT3, 2.15–4.12 pg/ml.

Thyroid disorders were defined using blood tests including TSH as well as fT4. TSH levels lower than the normal range of 0.44 μU/ml indicate hyperthyroidism, and TSH levels higher than the normal range of 3.77 μU/ml indicate hypothyroidism. Whether the dysfunction is clinically relevant is determined by thyroxine. If TSH levels are out of normal range but thyroxine levels are within the normal range, the thyroid disorder is classified as subclinical. If TSH is lower and thyroxine is higher than the normal range of 1.66 ng/dl, this indicates clinically relevant hyperthyroidism. If TSH is higher and thyroxine is lower than the normal range of 0.76 ng/dl, this indicates clinically relevant hypothyroidism.

### Statistical Analysis

Chi-square test was utilized for the analysis of the distribution of dichotomized variables. Due to the hypothesis generating design of the current study, no correction for multiple testing was applied. Patients without an event (death) were censored at the date that they were last known to be alive.

Differences between groups were assessed using the chi-square test, the Kruskal-Wallis test, the Mann-Whitney U test, and the Pearson correlation as appropriate.

OS was calculated from the date of the initial diagnosis to the death of the patient or the patient’s last follow-up date. Analyses of OS were done with Kaplan-Meier survival estimates with log-rank test and Cox regression. Using log-rank test, following parameters were correlated with outcome: gender, body mass index, nicotine, alcohol, family history, localization of cancer, histology, number of metastatic localizations, treatment (palliative chemotherapy vs best supportive care), gastrectomy, radiotherapy, second oncology, and known endocrinological disorders. Using Cox-regression analysis, following parameters were correlated with outcome: age, TSH, fT3, T3, T4, and fT4.

Two-tailed *p* values of ≤ 0.05 were considered to be statistically significant. All statistics were calculated using statistical package for the social sciences (SPSS) 24.0 software (SPSS Inc., Chicago, IL, USA).

## Results

About 258 patients with gastroesophageal cancer were included in further analysis. Table 1 shows the demographics and the baseline characteristics. Eighty patients (31%) were women, and the median age at diagnosis was 61 years (range of 27–91). About 117 (45%) patients were smokers, and 100 (38%) had a drinking habit. Interestingly, 51 patients (20%) had a positive family history for cancer, 27 (11%) of them even positive for gastroesophageal cancer.

**Table 1:** Patient demographic characteristics and their correlation with survival

Characteristic	Value	*p*	OS in days/HR (95% CI)
Gender [n (%)]		0.795	
Male	178 (69%)		297 (249–345)
Female	80 (31%)		275 (217–333)
Age (years)		0.096	
Median (SD)	61 (12.2)		1.009 (0.998–1.020)
Body mass index (BMI)		0.330	
Median (SD)	23.5 (4.4)		1.016 (0.984–1.049)
Nicotine [n (%)]		0.561	
No	141 (55%)		283 (215–351)
Yes	117 (45%)		286 (221–351)
Alcohol [n (%)]		0.268	
No	158 (62%)		276 (222–330)
In moderation	81 (31%)		348 (267–429)
Abuse	19 (7%)		229 (94–364)
Positive family history [n (%)]			
No	207 (80%)		278 (233–323)
Cancer in general	51 (20%)	0.526	335 (228–442)
Gastroesophageal cancer	27 (11%)	0.806	335 (258–412)

Table 2 shows the gastric cancer–specific characteristics and treatment modalities. In 124 (48%) patients, the tumor was located in the stomach, in 70 (27%) patients at the gastroesophageal junction and in 64 (25%) patients in the esophagus. There were 32 (12%) squamous cell carcinomas and 226 (88%) adenocarcinomas. Regarding metastasis, 150 patients (58%) had only one site of metastasis, 85 patients (33%) had two sites, and there were also patients with three [20 (8%)], four [2 (1%)], and five [1] sites of metastasis when they were first diagnosed. The most common metastatic sites were the liver, the peritoneum/omentum, and the lymph nodes with 106 patients (41%), 103 patients (40%), and 91 patients (35%), respectively. Around 10% of the patients had a second cancer either before or at the same time of the diagnosis of gastroesophageal cancer. Concerning treatment options, 228 (88%) patients received palliative chemotherapy to reduce the tumor load and relief symptoms; only 30 (12%) patients received best supportive care without any chemotherapy. Radiation therapy was used in 48 patients (19%), and 39 patients (15%) received a palliative gastrectomy.

**Table 2:** Characteristics of the malignant disease and its treatment and their correlation with survival

Characteristic	Value	*p*	OS in days/HR (95% CI)
Localization of cancer [n (%)]		0.518	
Gastric	124 (48%)		278 (224–332)
Gastroesophageal junction	70 (27%)		339 (263–415)
Esophageal	64 (25%)		286 (188–384)
Histological type [n (%)]		0.973	
Adenocarcinoma	226 (88%)		286 (240–332)
Squamous cell carcinoma	32 (12%)		284 (98–470)
Primary localization of metastasis [n (%)]		0.658*	
Liver	106 (41%)		351 (277–425)
Peritoneum/omentum	103 (40%)		250 (203–297)
Lymph nodes	91 (35%)		287 (196–378)
Lung	38 (15%)		364 (219–509)
Bones	22 (9%)		276 (136–416)
Muscle	9 (4%)		225 (169–281)
Pancreas	9 (4%)		541 (169–913)
Krukenberg tumor	4 (2%)		189 (96–282)
Adrenal glands	3 (1%)		276 (36–516)
Colon	3 (1%)		33 (31–35)
Brain	3 (1%)		265 (183–623)
Kidney	2 (1%)		220
Treatment [n (%)]		< 0.001	
Chemotherapy	228 (88%)		332 (289–375)
Best supportive care	30 (12%)		80 (55–105)
Palliative gastrectomy [n (%)]		0.117	0.736 (0.497–1.089)
Yes	39 (15%)		
Radiotherapy [n (%)]		0.738	
No	210 (81%)		283 (226–340)
Yes	48 (19%)		311 (246–376)
Second cancer [n (%)]		0.832	
No	72 (89%)		283 (227–339)
Yes	28 (11%)		297 (243–351)

*survival analysis performed comparing the number of metastatic sites

### Thyroid Disorders

Characteristics of patients with thyroid dysfunction are shown in Table 3. Around 61 patients (24%) of our study population had documented abnormal thyroid parameters, and 20 (8%) struggled with hyperthyroidism and 41 (16%) with hypothyroidism. Some patients had subclinical thyroid disorders, which means that only TSH levels are dysregulated, while fT4 levels remained within normal range. Around 15 patients had subclinical hyperthyroidism (75% of 20 hyperthyroid patients), and 14 patients had subclinical hypothyroidism (34% of 41 hypothyroid patients). The time of the diagnosis in patients with hyperthyroidism was mainly after the diagnosis of cancer (17 patients, 85%); only three patients had a known hyperthyroidism before the diagnosis of gastroesophageal cancer (one of them is subclinical hyperthyroidism). The time of the diagnosis in patients with hypothyroidism was mainly before they were diagnosed with cancer [26 (63%) vs 15 (37%) patients]. Only one of the hypothyroidisms before cancer diagnosis was subclinical; the other 13 cases of subclinical hypothyroidisms occurred after the cancer diagnosis.

**Table 3:** Characteristics of patients with thyroid dysfunction

		Hyperthyroidism (*n* = 20)	Hypothyroidism (*n* = 41)
Sex [n (%)]	Male	17 (85%)	21 (51%)
	Female	3 (15%)	20 (49%)
Classification [n (%)]	Clinical	5 (25%)	27 (66%)
	Subclinical	15 (75%)	14 (34%)
Time of onset [n (%)]	Before cancer diagnosis	3 (15%)	26 (63%)
	After cancer diagnosis	17 (85%)	15 (37%)
Treatment [n (%)]	Partial thyroidectomy	–	10* (24%)
	Hormone substitution	–	26 (63%)
	No treatment	19 (95%)	15 (37%)
	Thiamazole	1 (5%)	–

*one patient who had a partial thyroidectomy had no hypothyroidism afterward

About 26 patients, that is, 10% of the study population, received thyroid hormone replacement therapy at the time of their cancer diagnosis, and 11 patients, that is, 4% of the study population, had undergone partial thyroidectomy. All but one patient who had undergone partial thyroidectomy were hypothyroid after the procedure and had to take hormone replacement therapy. Only one out of the five patients with clinical hyperthyroidism received treatment for hyperthyroidism (thiamazole); the others did not get any treatment for this endocrinological disbalance.

Hypothyroidism was statistically significant diagnosed more often in women than in men (20/80 vs 21/178; *p* = 0.010). Other parameters like age (*p* = 0.130), tumor location (*p* = 0.536), histology (*p* = 0.192), family history (*p* = 1.000), second cancer (*p* = 0.412), smoking (*p* = 0.612), alcohol (*p* = 0.277), number of metastatic sites (*p* = 0.531), metastatic site (lymph nodes, *p* = 1.000; liver, *p* = 1.000; lung, *p* = 0.089; peritoneum, *p* = 0.863; bone, *p* = 1.000; Krukenberg, *p* = 0.502; kidney, *p* = 1.000; brain, *p* = 1.000; muscle, *p* = 0.362; pancreas, *p* = 0.638), and Her2 (*p* = 0.548) were not associated with hypothyroidism.

Hyperthyroidism was statistically significant diagnosed more often in patients with positive oncological family history (9/51 vs 11/207; *p* = 0.007) and in patients with liver (14/106 vs 6/152; *p* = 0.008) and lung metastases (7/38 vs 13/220; *p* = 0.016). Hyperthyroidism was not correlated with gender (*p* = 0.134), age (*p* = 0.328), tumor location (*p* = 0.691), histology (*p* = 0.484), second cancer (*p* = 0.051), nicotine (*p* = 0.100), alcohol (*p* = 0.878), number of metastatic sites (*p* = 0.160), metastatic sites (lymph nodes, *p* = 1.000; peritoneal, *p* = 0.094; bone, *p* = 0.392; Krukenberg, *p* = 1.000; renal, *p* = 1.000; brain, 0.216; muscle, 1.000; pancreatic, 1.000), or Her2 (*p* = 0.695).

### Thyroid Hormones

Thyroid parameters analyzed in this retrospective cohort including unit, normal range, median, standard deviation, minimum, and maximum are shown in Table 4**.**

**Table 4:** Thyroid parameters analyzed in this retrospective cohort including unit, normal range, median, standard deviation, minimum, and maximum

Thyroid parameter	Unit	Normal range	Median	Standard deviation	Minimum	Maximum
TSH	μU/ml	0.44–3.77	1.41	1.58	0.02	11.90
T3	ng/ml	0.8–1.8	1.01	0.26	0.49	1.53
T4	ng/ml	58–124	88.50	22.06	51.00	154.00
fT3	pg/ml	2.15–4.12	2.50	0.78	0.95	5.91
fT4	ng/dl	0.76–1.66	1.30	0.29	0.48	2.50

**Fig. 1:**
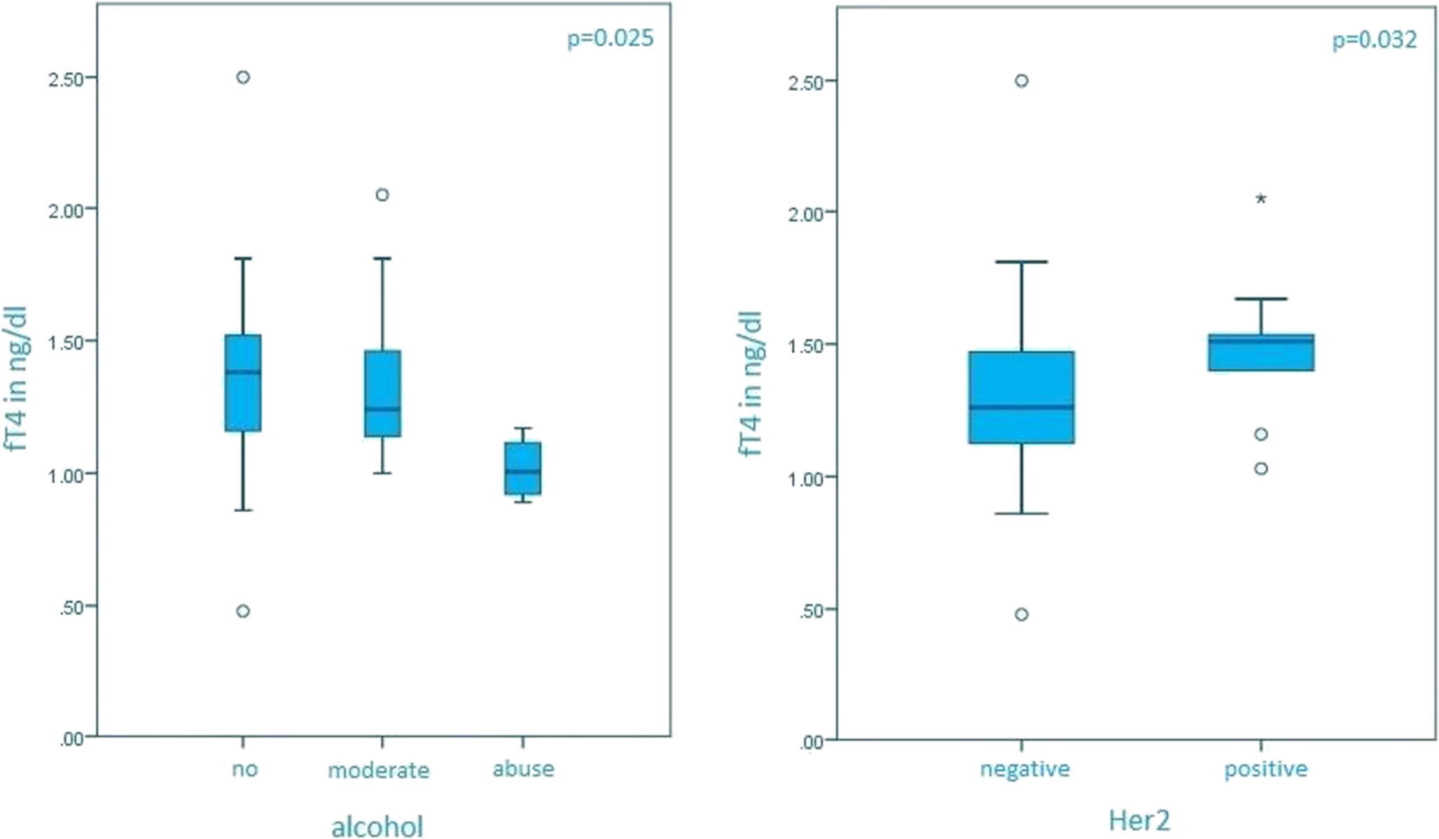
Correlation of fT4 with alcohol and Her2 positivity

fT4 levels were statistically significant correlated with Her2 status (*p* = 0.032, Her2-positive median of 1.51 ng/dl, Her2-negative median of 1.26 ng/dl) and alcohol (*p* = 0.025, no alcohol median of 1.38 ng/dl, moderate alcohol use median of 1.24 ng/dl, alcohol abuse median of 1.01 ng/dl). These results are shown in Fig. 1**.** fT4 levels did not correlate with age (*p* = 0.632; rho = −0.053), gender (*p* = 0.961), histology (*p* = 0.477), tumor localization (*p* = 0.), family history (*p* = 0.212), second cancer (*p* = 0.534), nicotine (*p* = 0.488), number of metastatic sites (*p* = 0.754), metastatic site (lymph nodes, *p* = 0.694; liver, *p* = 0.331; lung, *p* = 0.429; peritoneum, *p* = 0.206; bone, *p* = 0.070; Krukenberg, *p* = 0.434; kidney, *p* = 0.554; brain, *p* = 0.771; muscle, 0.640).

### Survival Analyses

The median overall survival (OS) of the study population was 286 days (95% CI 238–334).

The correlation of demographics and the baseline characteristics as well as characteristics of the malignancy and its treatment with survival are shown as *p* values in Tables 1 and 2.

### Correlation Thyroid Parameters and Survival

The TSH level did not significantly impact the survival outcome, but a trend could be seen that higher TSH levels often correlate with shorter survival (*p* = 0.070, HR = 1.102). Lower levels of T3 (*p* = 0.003, HR = 0.141) as well as higher levels of fT4 (*p* = 0.041, HR = 2.202) led to a significantly shorter survival. Different levels of T4 (*p* = 0.071, HR = 0.990) and fT3 (*p* = 0.938, HR = 0.986) had no influence on the overall survival.

**Fig. 2:**
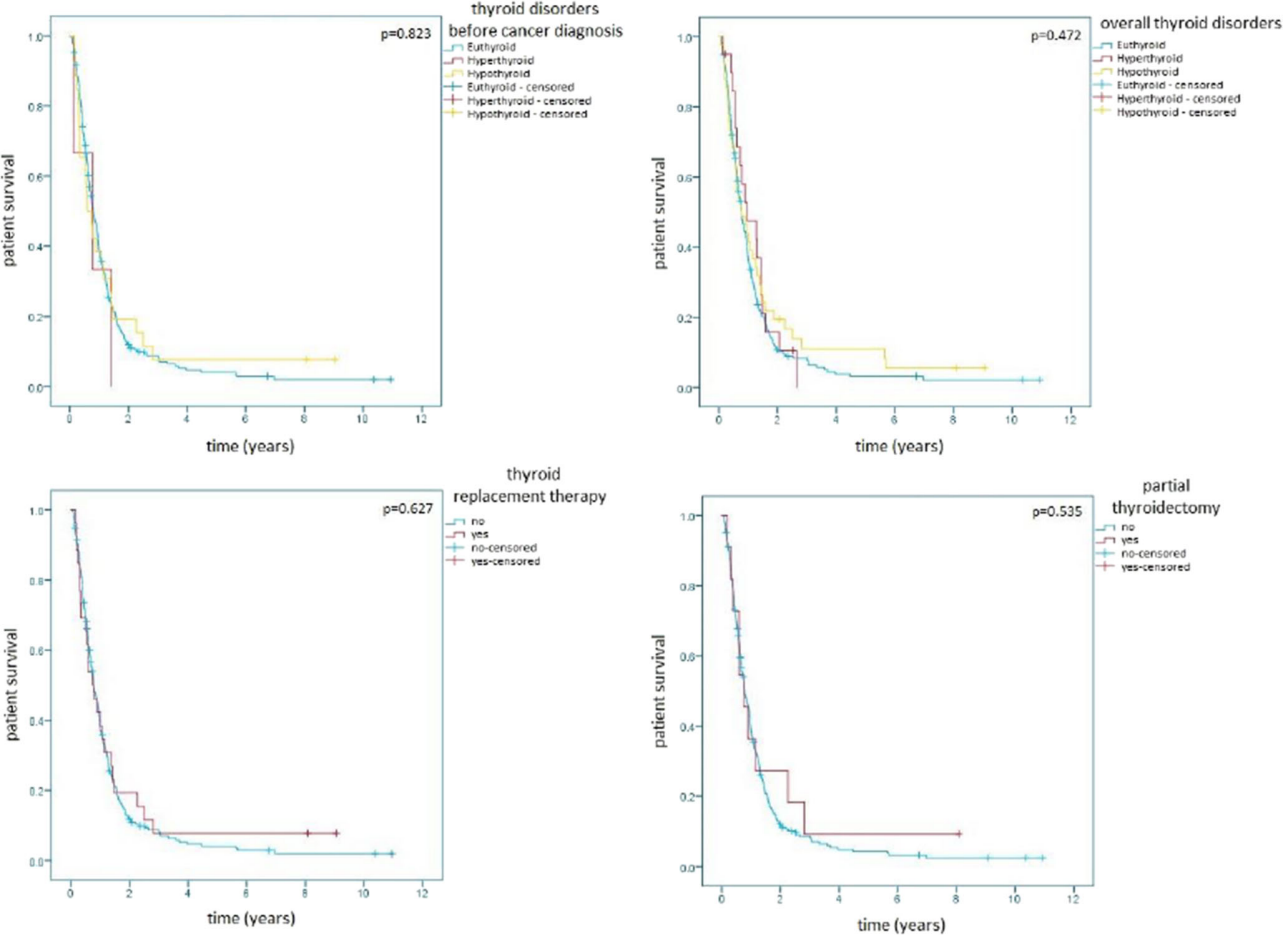
Kaplan-Meier survival curve of thyroid disorders and their therapies

The overall survival of patients with thyroid disorders did not differ significantly from euthyroid patients, neither considering all thyroid disorders throughout patient history (euthyroid, median OS of 283 days, 95% CI of 238–328; hyperthyroid, median OS of 354 days, 95% CI of 94–614; hypothyroid, median OS of 284 days, 95% CI of 122–446; *p* = 0.472) nor considering only thyroid disorders known before the diagnosis of cancer (euthyroid, median OS of 288 days, 95% CI of 238–338; hyperthyroid, median OS of 286 days, 95% CI of 0–665; hypothyroid, median OS of 218 days, 95% CI of 119–317; *p* = 0.823). The use of thyroid replacement therapy (without, median OS of 286 days, 95% CI of 234–338; with, median OS of 275 days, 95% CI of 138–412; *p* = 0.627) as well as partial thyroidectomy (without, median OS of 286 days, 95% CI of 235–337; with, median OS of 275 days, 95% CI of 156–394; *p* = 0.535) did not impact the survival outcome significantly. The Kaplan-Meier curves of these results are shown in Fig. 2.

## Discussion

The association of thyroid function and cancer progression has been addressed by several preclinical and epidemiological studies; however, to our best knowledge, we are the first to investigate the prognostic impact of thyroid function in a large cohort of patients suffering from metastatic gastroesophageal cancer. The most important finding of this analysis is that high fT4 levels, which fit a hyperthyroid state, indicate a poorer overall survival of patients with gastroesophageal cancer in an advanced stage. These results match the hypothesis that thyroid hormones modulate proliferative, metabolic, and angiogenic pathways and thereby lead to tumor progression [6, 13]. Our observation is well in line with previous preclinical and epidemiological studies surmising the conducive effects of high fT4 levels on tumor progression [11, 14–16]. Thus, further preclinical studies identifying potential therapeutic targets in the interaction of thyroid hormones and cancer cells are warranted.

Multiple preclinical as well as epidemiological investigations indicate that higher TSH levels are associated with a lower risk for specific types of cancer (such as prostate cancer, lung cancer, and breast cancer) and that higher fT4 levels are linked with an increased risk for the incidence and progression of these malignancies [11, 14–22]. Since patients with hypothyroidism were shown to be older at the diagnosis of breast cancer, it was surmised that the hypothyroid state actually might slow down tumor development and progression [8, 23].

Contrariwise, there are studies showing protective effects of thyroid hormones [24, 25]. Eventually, no direct association between thyroid hormones and cancer in general could be found as the results of multiple studies concerning this issue have not been consistent and so far a specific underlying pathophysiological mechanism has not been detected in vivo. Especially in patients with gastrointestinal cancer, comprehensive analyses of the prognostic impact of thyroid function are so far missing.

Thus, the aim of this study was to investigate the prognostic impact of endocrinological parameters, especially thyroid hormones, in a large, well-defined cohort of patients suffering from metastatic gastroesophageal cancer.

Interestingly, our study does not show any survival benefits for patients who were diagnosed with hypothyroidism, as well as no survival detriment for patients who were diagnosed with hyperthyroidism, neither before their cancer diagnosis nor throughout their entire patient history, respectively. Even though hyperthyroidism could be statistically significant correlated with liver and lung metastases suggesting an extensive malignant disease, no correlation of hyperthyroidism and OS could be shown.

Several studies show that the presence of hypothyroidism evolving during the application of chemotherapy is a marker of remission, improved treatment response, and decreased tumor growth in specific tumor types such as melanoma, renal cell carcinoma, gliomas, and indolent breast cancer [26]. This was not the case in our study, since hypothyroidism did not benefit the survival of this patient subgroup. However, one can surmise that chemotherapy might be a risk factor for thyroid disorders, since more than 12% of our study population developed hyperthyroidism (17 patients) or hypothyroidism (15 patients) during the course of cancer therapy. This assumption is in line with the findings of other trials [22, 26–28].There were also no changes of survival outcomes in correlation with the treatment of thyroid disorders.

Higher fT4 levels were associated with impaired survival prognosis in our series of patients suffering from metastatic gastric cancer suggesting that a rather hypothyroid state could impact the survival positively. In line, previous studies suggested that induced hypothyroidism could lead to a survival benefit of tumor patients, yet this effect could not be shown in randomized studies so far [6, 13].

Furthermore, a possible explanation for the associations of high fT4 as well as low T3 levels with the median overall survival in some patients might be the nonthyroidal illness syndrome (NTIS). This syndrome is a constellation of changes in circulating thyroid hormone levels that occur in euthyroid patients with chronic systemic diseases. Usually it is associated with a reduction in serum T3 and variable changes in serum fT4 levels. Although NTIS was believed to play no role in the development and proliferation of cancer cells, a recent review by Hercbergs et al. cited multiple studies that indicate an association between NTIS and malignant tumors [29]. The findings of our study suggest that NTIS might not be benign in the context of fT4 and gastroesophageal cancer. However, further studies in a prospective setting are needed to verify these results. If this hypothesis holds true, induced hypothyroxinemia (suppression of fT4 as well as the administration of T3) might be considered for patients with advanced cancers to whom other avenues of treatment are closed. This potential treatment option was already discussed by Hercbergs et al. [30].

Interestingly, in our cohort, fT4 levels could be associated with alcohol and Her2 positivity. Alcohol has been reported to cause direct suppression of thyroid function, which is in line with our findings [31]. However, the correlation of Her2 positivity and fT4 has not been described yet. Since Her2 was initially associated with a worse prognosis, which has only changed due to the establishment of treatment with trastuzumab, this finding may suggest that Her2 positivity and elevated fT4 levels collaborate on tumor progression [32, 33]. However, the exact mechanism of thyroid hormones as driver for cancer progression as well as a prognostic factor is still not known precisely, thus preventing development of new treatment options. Therefore, a deeper investigation of the underlying molecular mechanisms might allow the identification of promising new treatment targets. The results of our study strongly support the further preclinical investigation of pathways activated by thyroid hormones as potential targets for cancer therapy [13].Furthermore, an association of nicotine abuse and alterations of thyroid hormones has been described in literature [34]. In this analysis, neither hyperthyroidism nor hypothyroidism or fT4 levels could be correlated with smoking. However, since this topic is highly debated, further prospective studies might bring more insight into this association and might correlate the results with the overall survival of cancer patients.

Strengths and limitations of the study need to be considered. The patient population was homogenous: all patients had metastatic gastroesophageal cancer at presentation and were therefore in a palliative treatment setting with an estimated survival of about 9 to 12 months. All patients were treated according to the individual decision of an interdisciplinary tumorboard, which ensured the best possible treatment according to the respective standard of knowledge at the time of diagnosis. All patients were followed up regularly.

The correlations of endocrinological parameters with survival were analyzed using the Cox regression, since this form of testing provides better estimates of survival probabilities and cumulative hazard than those provided by the Kaplan-Meier function. One important limitation of this study, as it is a retrospective analysis, is missing data concerning the laboratory results. Since regularly routine laboratory monitoring during the treatment with chemotherapy is obligatory and standardized at the Vienna General Hospital, most results were retrievable from the medical records. However, there were still missing endocrinological parameters in some patients. Data was obtained in 180 patients considering TSH, 73 considering fT3, 49 considering T3, 78 considering T4, and 83 considering fT4. Furthermore, in this study, laboratory results were only analyzed at the time of cancer diagnosis. Further treatment strategies as well as other interventions might influence the longitudinal effects of thyroid hormones and thereby influence the overall survival. These sequential laboratory parameters were not retrieved and therefore not discussed in this paper. Thus, to confirm the results of this study, a prospective study should be conducted to minimize missing data points. Furthermore, a more detailed examination of laboratory values concerning thyroid hormones, including the presence of autoimmune antibodies as well as thyroid hormone receptors, in a prospective cohort study would be of interest to identify the underlying cause of the observed association between a hyperthyroid state and a poorer survival prognosis. Since our analyses were performed at the time of cancer diagnosis, the investigation of the longitudinal effects of endocrinological parameters on cancer development is an important objective in future studies.

In conclusion, in this retrospective single center analysis, high fT4 and low T3 levels indicate a poorer overall survival of patients with gastroesophageal cancer in an advanced stage. Since data on the correlation of endocrinological parameters and gastroesophageal cancer are scarce, this analysis is an important impulse for further studies concerning the impact of hormones on patients with tumors of the upper GI tract.

## Data Availability

The data that support the findings of this study are available from the corresponding author, A.I-M., upon reasonable request.

## Abbreviations

BMI: Body mass index
fT3: Free triiodothyronine
fT4: Free thyroxine
GI: Gastrointestinal
NTIS: Nonthyroidal illness syndrome
OS: Overall survival
SD: Standard deviation
T3: Triiodothyronine
T4: Thyroxine
TSH: Thyroid-stimulating hormone
vs: Versus
